# Ibrutinib: Implications for Use in the Treatment of Mantle Cell Lymphoma and Chronic Lymphocytic Leukemia

**DOI:** 10.6004/jadpro.2015.6.5.3

**Published:** 2015-09-01

**Authors:** Gretchen Anne McNally, Jennifer M. Long, Lynne R. Brophy, Maria R. Badillo

**Affiliations:** 1The Ohio State University–Arthur G. James Cancer Hospital, Columbus, Ohio; 2Whittingham Cancer Center, Norwalk Hospital, Norwalk, Connecticut; 3TriHealth Cancer Institute, Cincinnati, Ohio; 4MD Anderson Cancer Center, Houston, Texas

**Ibrutinib: Implications for Use in the Treatment of Mantle Cell Lymphoma and Chronic Lymphocytic Leukemia**

A continuing education article for nurse practitioners, physician assistants, clinical nurse specialists, advanced degree nurses, oncology and hematology nurses, pharmacists, and physicians.

**Release date:** September 15, 2015

**Expiration date:** September 15, 2016

**Expected time to complete this activity as designed:** 0.50 hours

**Meniscus Educational Institute**

3131 Princeton Pike,

Building 1, Suite 205A

Lawrenceville, NJ 08648

Voice: 609-246-5000

Fax: 609-449-7969

E-mail: lrubin@meniscusedu.com

**Journal of the Advanced Practitioner in Oncology**

37 Main Street

Cold Spring Harbor, NY 11724

Voice: 631-692-0800

Fax: 631-692-0805

E-mail: claudine@harborsidepress.com

© *2015, Meniscus Educational Institute. All rights reserved.*

## Faculty

**Gretchen Anne McNally, PhD, ANP-BC,** The Ohio State University–Arthur G. James Cancer Hospital

**Jennifer M. Long, APRN,** Whittingham Cancer Center, Norwalk Hospital

**Lynne R. Brophy, RN,** TriHealth Cancer Institute

**Maria R. Badillo, MSN, RN,** University of Texas MD Anderson Cancer Center

## Activity Rationale and Purpose

The purpose of this article is to provide advanced practitioners with important information about new treatment options for mantle cell lymphoma and chronic lymphocytic leukemia, including novel oral agents, management of related side effects, and patient education.

## Intended Audience

The activity’s target audience will consist of nurse practitioners, physician assistants, clinical nurse specialists, advanced degree nurses, oncology and hematology nurses, pharmacists, and physicians.

## Learning Objectives

After completing this educational activity, participants should be able to:

1. Identify clinical features and considerations to determine which patients are eligible for treatment with ibrutinib
2. Recognize potential side effects and adverse events associated with ibrutinib and intervene to prevent, assess, and/or manage them appropriately should they occur

## Continuing Education

**Statement of Credit—Participants who successfully complete this activity (including the submission of the post-test and evaluation form) will receive a statement of credit.**

**Physicians.** The Meniscus Educational Institute is accredited by the Accreditation Council for Continuing Medical Education (ACCME) to provide continuing medical education for physicians.

The Meniscus Educational Institute designates this journal article for a maximum of *0.50 AMA PRA Category 1 Credits*™. Physicians should claim only the credit commensurate with the extent of their participation in the activity.

**Nurses.** This activity for 0.50 contact hours is provided by the Meniscus Educational Institute.

The Meniscus Educational Institute is accredited as a provider of continuing nursing education by the American Nurses Credentialing Center’s Commission on Accreditation.

Provider approved by the California Board of Registered Nursing, Provider No. 13164, for 0.50 contact hours.

**Pharmacists.** The knowledge-based accredited education lectures are intended for pharmacists involved in the care of cancer patients. This educational activity is sponsored by the Meniscus Educational Institute.

The Meniscus Educational Institute is accredited by the Accreditation Council for Pharmacy Education (ACPE) as a provider of continuing pharmacy education. The ACPE Universal Activity Number assigned to this program, for 0.50 contact hours, is 0429-9999-15-015-H01-P.

## Financial Disclosures

All individuals in positions to control the content of this program (eg, planners, faculty, content reviewers) are expected to disclose all financial relationships with commercial interests that may have a direct bearing on the subject matter of this continuing education activity. Meniscus Educational Institute has identified and resolved all conflicts of interest in accordance with the MEI policies and procedures. Participants have the responsibility to assess the impact (if any) of the disclosed information on the educational value of the activity.

**Faculty**

**Gretchen Anne McNally, PhD, ANP-BC,** has acted as a consultant and served on speakers bureuas for Pharmacyclics.

**Jennifer M. Long, APRN,** has been a member of speakers bureaus for Celgene, Lilly, and Pfizer.

**Lynne R. Brophy, RN,** has owned stock in Amgen and Johnson & Johnson.

**Maria R. Badillo, MSN, RN,** has nothing to disclose.

**Lead Nurse Planner**

**Wendy J. Smith, ACNP, AOCN®,** has nothing to disclose.

**Planners**

**Jeannine Coronna** has nothing to disclose.

**Claudine Kiffer** has nothing to disclose.

**Terry Logan, CHCP,** has nothing to disclose.

**Pamela Hallquist Viale, RN, MS, CNS, ANP,** has nothing to disclose.

**Lynn Rubin** has nothing to disclose.

**Content Reviewers**

**Glenn Bingle, MD, PhD, FACP,** has nothing to disclose.

**Kate D. Jeffers, PharmD, BCOP,** has nothing to disclose.

**Margaret Fields, RN, ACNP-BC, AOCNP®,** has nothing to disclose.

**Wendy J. Smith, ACNP, AOCN®,** has nothing to disclose.

## Disclaimer

This activity has been designed to provide continuing education that is focused on specific objectives. In selecting educational activities, clinicians should pay special attention to the relevance of those objectives and the application to their particular needs. The intent of all Meniscus Educational Institute educational opportunities is to provide learning that will improve patient care. Clinicians are encouraged to reflect on this activity and its applicability to their own patient population.

The opinions expressed in this activity are those of the faculty and reviewers and do not represent an endorsement by Meniscus Educational Institute of any specific therapeutics or approaches to diagnosis or patient management.

## Product Disclosure

This educational activity may contain discussion of published as well as investigational uses of agents that are not approved by the US Food and Drug Administration. For additional information about approved uses, including approved indications, contraindications, and warnings, please refer to the prescribing information for each product.

## How to Earn Credit

To access the learning assessment and evaluation form online, www.meniscusce.com

**Statement of Credit:** Participants who successfully complete this activity (including scoring of a minimum of 70% on the learning assessment and complete and submit the evaluation form with an E-mail address) will be able to download a statement of credit.

## ABSTRACT

Bruton’s tyrosine kinase (BTK) is expressed in B-cell malignancies, playing an important role in B-cell receptor (BCR) signaling and offering a promising new strategy for the development of targeted drugs. Malignant B cells in mantle cell lymphoma (MCL) and chronic lymphocytic leukemia (CLL) rely on BCR signaling pathways for cell survival, proliferation, adhesion, and migration. Ibrutinib, a first-in-class orally bioavailable, small-molecule inhibitor of BTK, was approved in the United States for the treatment of patients with relapsed or refractory MCL and CLL, as well as patients with CLL who have deletion 17p. Ibrutinib has been shown to prevent proliferation and induce apoptosis of malignant B cells while also blocking cellular responses to survival stimuli from the tumor microenvironment. Ibrutinib has a favorable risk-benefit profile and is effective in patients with relapsed or refractory MCL and CLL, for whom treatment options are limited. Advanced oncology providers play a critical role in explaining the mechanism of action of this novel oral agent, educating patients and caregivers on successful self-administration of ibrutinib within the clinical setting, as well as monitoring and managing potential side effects.

## ARTICLE

Novel agents that target B-cell receptor (BCR) signaling pathways have afforded new treatment options for patients with B-cell malignancies, providing important clinical benefits in a number of hematologic tumor types. Ongoing studies continue to evaluate the safety and effectiveness of these agents. Here, we focus on one such targeted agent, the Bruton’s tyrosine kinase (BTK) inhibitor ibrutinib (Imbruvica), and implications for its use in the treatment of mantle cell lymphoma (MCL) and chronic lymphocytic leukemia (CLL).

Ibrutinib is a highly active new agent that is safe and effective in a heavily pretreated and older adult population (Byrd et al., 2014a; Wang et al., 2013). On January 29, 2015, the US Food and Drug Administration (FDA) announced ibrutinib was the first drug approved for the treatment of Waldenström’s macroglobulinemia. The use of ibrutinib in patients with Waldenström’s macroglobulinemia is not discussed further in this article, as very little clinical data was available at the time this article was written.

## Overview of MCL and CLL

MCL is a rare yet well-defined subtype of B-cell lymphoma, accounting for 5% to 10% of non-Hodgkin lymphomas (NHLs; Swerdlow, Campo, Seto, & Muller-Hermelink, 2008; Zaja, Federico, Vitolo, & Zinzani, 2014). Patients with MCL have a median age of 60 to 65 years, with a male predominance of 2:1 (Swerdlow et al., 2008; Zaja et al., 2014).

Patients with MCL typically have advanced-stage disease, extensive lymphadenopathy, splenomegaly, and bone marrow involvement, with or without peripheral blood involvement. Extranodal sites often include the gastrointestinal tract (Swerdlow et al., 2008). Mantle cell lymphoma is incurable with standard therapy and has a poor prognosis and an aggressive clinical course characterized by resistant and relapsing disease (Zaja et al., 2014). In November 2013, ibrutinib was approved by the FDA as single-agent therapy in patients with MCL after at least one prior therapy (Pharmacyclics, 2014).

Chronic lymphocytic leukemia is the most commonly diagnosed leukemia in adults, with a reported median age at diagnosis of 71 years (Howlader et al., 2013; Muller-Hermelink et al., 2008). In 2013, CLL was diagnosed in nearly 16,000 persons and caused more than 4,500 deaths (Howlader et al., 2013). Chronic lymphocytic leukemia usually affects the peripheral blood and bone marrow, as well as the lymph nodes, liver, and spleen; small lymphocytic leukemia (SLL) is considered part of the same entity, manifesting primarily in the lymph nodes and spleen without peripheral lymphocytosis (Muller-Hermelink et al., 2008).

Chronic lymphocytic leukemia remains incurable with current therapies and is generally associated with an indolent disease course in patients with favorable prognostic factors (median survival, 293 months). Patients with high-risk cytogenetics, including deletion of the short arm of chromosome 17 (del 17p), have a more aggressive course and an inferior prognosis than those without this abnormality (Hallek, 2013; Muller-Hermelink et al., 2008). Treatment is typically deferred ("watch and wait") until clinical symptoms develop, indicating a need for therapy (i.e., bulky lymphadenopathy and/or splenomegaly, cytopenias, fevers without infection, drenching night sweats, profound fatigue, significant unexplained weight loss). Factors considered in treatment decisions include age, performance status, comorbidities, cytogenetics, and the therapeutic goal (disease control or palliation).

Standard chemoimmunotherapy is not curative, and options for relapsed or refractory CLL are often associated with increased toxicity (O’Brien et al., 2014). The need to improve outcomes in older patients or those with high-risk disease remains. Ibrutinib, an oral agent with a novel mechanism of action, was approved by the FDA as single-agent therapy for patients with relapsed or refractory CLL and patients with CLL who have del 17p, including both those who are treatment-naive and those who have received prior therapy.

## Background on BTK

External signals from the microenvironment are critical to B-cell malignancy development and survival. The dependence of malignant B cells on these signals is complex and highly variable. Genetic abnormalities allow for a proliferation advantage, whereas dysfunctional microenvironments provide growth and drug-resistance signals. Apoptosis may be prevented under such circumstances (Burger, Ghia, Rosenwald, & Calagaris-Cappio, 2009).

In 1952, Colonel Ogden Bruton diagnosed a lack of gamma globulins in a young boy with primary immunodeficiency disease. The causative gene of X-linked agammaglobulinemia was identified in 1993 and named Bruton agammaglobulinemia tyrosine kinase (Khan, 2012). An important kinase, BTK is positioned early in the BCR signaling pathway and plays a critical role in the development, proliferation, apoptosis, and other cellular processes of normal B cells. A number of B-cell malignancies overexpress BTK, causing dysregulation of usual activities and making it a potential therapeutic target (Chung & Lee, 2014; Robak & Robak, 2012).

Ibrutinib forms an irreversible covalent bond to cysteine 481, which blocks B-cell activation and signaling. This process prevents proliferation, promotes apoptosis, and stops the malignant cells’ response to prosurvival stimuli in the microenvironment (Chung & Lee, 2014; Robak & Robak, 2012). Ibrutinib also inhibits several other kinases, unlike idelalisib (Zydelig), another oral BCR inhibitor, which is a selective reversible inhibitor of PI3Kẟ (Byrd, Jones, Woyach, Johnson, & Flynn, 2014b).

## Clinical Trial Data

A phase I dose-escalation study of ibrutinib in 56 patients with relapsed/refractory B-cell malignancies (NHL, CLL, SLL, or Waldenström’s macroglobulinemia) demonstrated an overall response rate (ORR) of 60% (including 16% complete response) in 50 evaluable patients, with grade 3 or 4 side effects occurring infrequently. The highest ORR was reported in patients with MCL (7 of 9, 78%) and CLL (11 of 16, 69%; Advani et al., 2013). Median progression-free survival (PFS) was 13.6 months for all patients in this study.

Two different dosing schedules were examined, both using once-daily administration; for 28 consecutive days, this regimen was followed by 7 days off (35-day cycle) or continuous dosing until disease progression or unacceptable toxicity. The maximum tolerated dose was not reached. This study indicated that continuous once-daily ibrutinib dosing has a tolerable safety profile and may be needed to maintain optimal antitumor effects. Thus, continuous dosing was recommended for subsequent phase II ibrutinib studies.

Breakthrough Therapy Designation is part of the 2012 FDA Safety and Innovation Act intended to expedite the development and review of new drugs for serious or life-threatening diseases. For the FDA to grant this designation, preliminary clinical evidence must show that the drug demonstrates substantial improvement over existing therapies on one or more clinically significant endpoints (FDA, 2013).

Breakthrough Therapy Designation for ibrutinib in patients with relapsed or refractory MCL was granted in February 2013 (FDA, 2013), based on an international, multicenter, open–label phase II study evaluating the efficacy and safety of ibrutinib in patients with heavily pretreated MCL (median 3 prior therapies). Patients enrolled in this study included those with (n = 50, 48 treated) and without (n = 65, 63 treated) prior bortezomib treatment. All treated patients (n = 111) received a fixed, once-daily oral dose of ibrutinib (560 mg). Treatment was continued until disease progression or unacceptable toxicity was noted (Wang et al., 2013). Key findings at median follow-up of 15.3 months included a 68% ORR (including 21% complete response) and a 17.5-month median duration of response (Table 1).

**Table 1:**
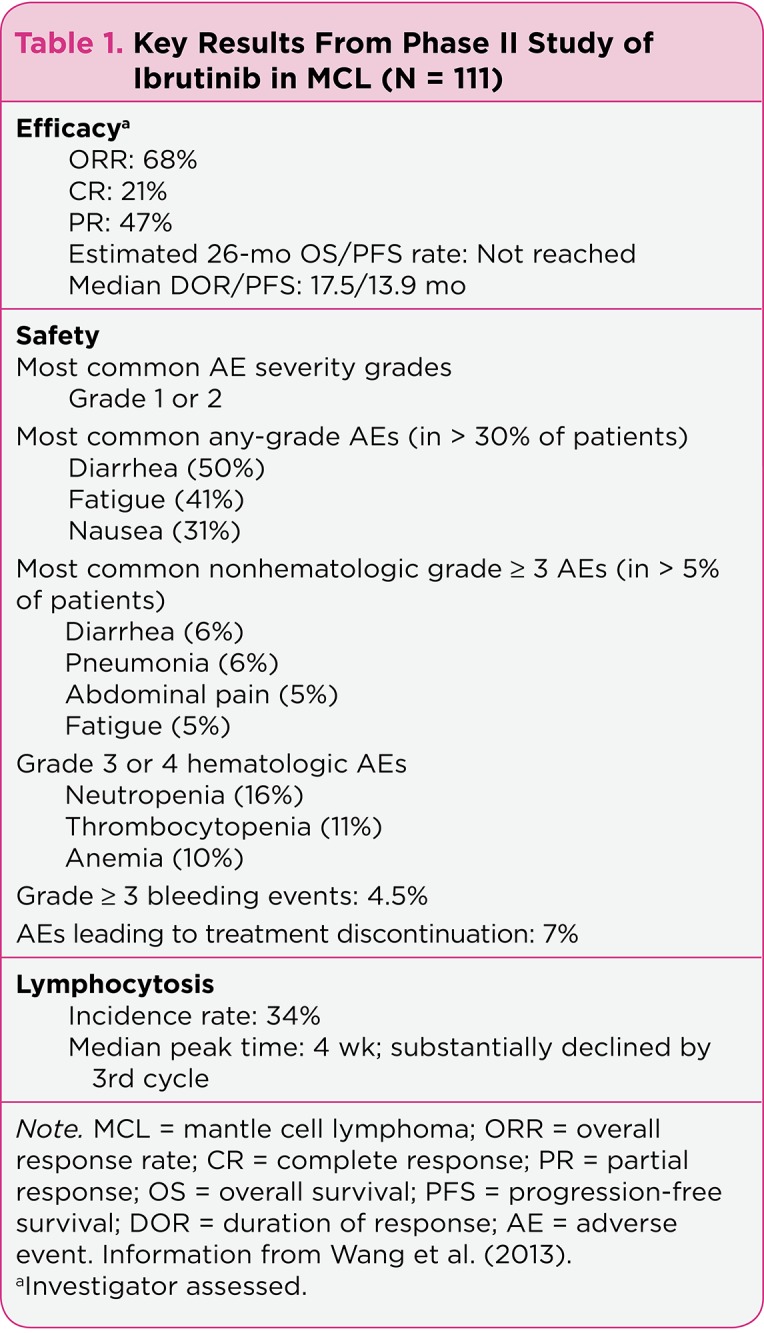
Key Results From Phase II Study of Ibrutinib in MCL (N = 111)

The Breakthrough Therapy Designation for ibrutinib in patients with CLL/SLL with del 17p was granted in April 2013 (FDA, 2013), based on a phase Ib/2 multicenter, open-label study evaluating the safety, efficacy, pharmacokinetics, and pharmacodynamics of ibrutinib. Results from the RESONATE trial, a multicenter, open-label, phase III study, were released in June 2014.

The RESONATE trial randomized patients (N = 391) with relapsed or refractory CLL to daily ibrutinib or ofatumumab (Arzerra), the anti-CD20 antibody. At a median follow-up of 9.4 months, ibrutinib significantly improved PFS (88% at 6 months), and the median duration of response was not reached (Byrd et al., 2014a). The median PFS was 8.1 months in the ofatumumab group. The OS at 12 months was 90% for the ibrutinib group compared with 81% in the ofatumumab group. All responses were partial, as there were no complete responses, and an additional 20% of the patients had a partial response with lymphocytosis. Lymphocytosis is recognized as a class effect of BCR-targeting agents. Ibrutinib was superior to ofatumumab in PFS and OS in all subgroups, including del 17p, resistance to previous purine analog therapy, age, and prior treatment regimens (Byrd et al., 2014a; Table 2).

**Table 2:**
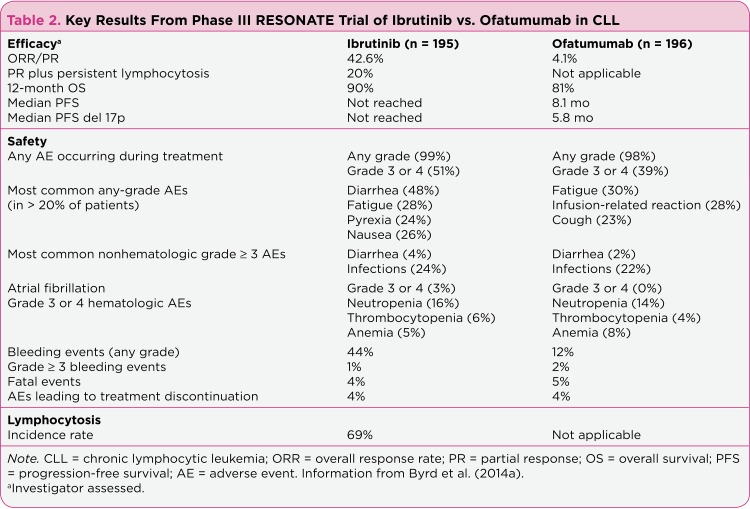
Key Results From Phase III RESONATE Trial of Ibrutinib vs. Ofatumumab in CLL

Few patients have progressed while taking ibrutinib, and ibrutinib relapse often occurs in the setting of Richter’s transformation and less frequently in CLL progression (Byrd et al., 2014b). Understanding the resistance mechanism is important for developing successful salvage therapies.

Studies suggest the primary mutation *C481S* in BTK prevents the drug from covalent, irreversible drug binding. Three mutations were also discovered in *PLCy2*, including *S707Y*, *R665W*, and *L845F* (Woyach et al., 2014a). Patients with more genomic instability, such as del 17p or a complex karyotype, may be at higher risk for developing resistance to ibrutinib. Most likely other mechanisms of resistance are also present, and this topic is currently being explored further (Woyach et al., 2014a). Byrd et al. (2014b) reported that discontinuing ibrutinib therapy may result in rapid disease progression in relapsing patients, and instead they recommended continuing ibrutinib therapy until immediately before the next treatment.

**Dosing and Administration**

Ibrutinib dosing for patients with MCL is 560 mg (four 140-mg capsules) orally once daily, whereas dosing for patients with CLL is 420 mg (three 140-mg capsules) orally once daily. The capsules should not be opened, broken, or chewed and should be taken with a glass of water at approximately the same time each day. If a dose is missed, it should be taken as soon as possible on the same day, and the patient should return to the normal schedule the next day. If a dose is accidentally skipped, extra capsules should not be taken.

Ibrutinib therapy should be interrupted for any grade ≥ 3 nonhematologic toxicity, grade ≥ 3 neutropenia with infection or fever, or grade 4 hematologic toxicities. Once the symptoms of the toxicity have resolved to grade 1 or baseline (recovery), ibrutinib therapy may be reinitiated. Recommended dose modifications for these toxicities are shown in Table 3 (Pharmacyclics, 2014).

**Table 3:**
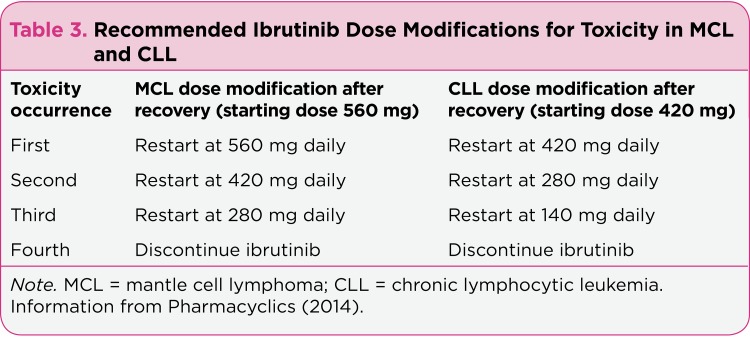
Recommended Ibrutinib Dose Modifications for Toxicity in MCL and CLL

Ibrutinib is primarily metabolized in the liver by CYP3A. Ibrutinib exposure data for patients with impaired hepatic function are not currently available. Thus, its use should be avoided in patients with baseline hepatic impairment (Pharmacyclics, 2014).

Examples of moderate CYP3A inhibitors are ciprofloxacin, diltiazem, fluconazole, and verapamil, among others. Grapefruit juice and Seville oranges, which are known to inhibit CYP3A, should also be avoided. Strong inducers of CYP3A can decrease the concentration of ibrutinib by approximately tenfold; thus, coadministration of CYP3A inducers should be avoided. Such agents include carbamazepine, rifampin, phenytoin, and St. John’s wort (Pharmacyclics, 2014; U.S. FDA, 2014). Patients should be advised to inform their health-care provider of all concomitant medications, including prescription and over-the-counter drugs, vitamins, and herbal products.

Interruption of ibrutinib therapy should be considered for short-term use of strong CYP3A inhibitors (e.g., antifungals or antibiotics such as voriconazole or clarithromycin for < 7 days). If chronic coadministration of moderate CYP3A inhibitor is necessary, the dose of ibrutinib should be decreased to 140 mg daily (1 capsule), and patients should be closely monitored for symptoms of ibrutinib toxicity.

**Lymphocytosis**

Ibrutinib causes a rapid decrease in lymphadenopathy, and a simultaneous shift of lymphocytes to the peripheral blood results in transient lymphocytosis (Byrd et al., 2013; Wang et al., 2013). Inhibition of BTK may also impair adhesion of B cells in the bone marrow and nodal sites, potentially contributing to the mobilization of malignant cells to blood (Advani et al., 2013; de Rooij et al., 2012; Woyach et al., 2014b). Patients with MCL who develop lymphocytosis (absolute lymphocyte count > 400,000/µL) have developed intracranial hemorrhage, lethargy, gait instability, and headache, although some of these cases were in the setting of disease progression (Pharmacyclics, 2014).

Clinical studies with ibrutinib reported lymphocytosis in 77% of CLL patients, with the onset of isolated lymphocytosis occurring during the first month of therapy and resolving by a median of 23 weeks (Pharmacyclics, 2014). In contrast, a smaller percentage of MCL patients developed lymphocytosis (33%), with the onset of isolated lymphocytosis occurring during the first few weeks of therapy and resolving by a median of 8 weeks (Pharmacyclics, 2014). Lymphocytosis in the setting of improvement in other disease parameters should not be considered treatment failure or progressive disease in patients receiving a BCR-targeting agent (Hallek et al., 2012). A landmark analysis evaluating patients with persistent lymphocytosis at 1 year and patients who achieved responses without lymphocytosis found similar PFS benefits in both groups (Woyach et al., 2014b).

**Precautions**

Hemorrhagic events (ranging from petechiae and bruising to intracranial hemorrhage) have been reported in patients treated with ibrutinib, regardless of platelet counts. Of 111 patients with MCL treated with ibrutinib, 4 had subdural hematomas (all grade ≤ 3) associated with falls, head trauma, or both. These patients also had received either aspirin or warfarin therapy within 2 days of the bleeding event (Wang et al., 2013). The RESONATE study excluded patients requiring warfarin but not other forms of anticoagulation. Major hemorrhage was similar between the two study groups, with one subdural hematoma noted in a patient receiving ibrutinib. Mild bleeding episodes were more common in the ibrutinib group (Byrd et al., 2014a).

Patients should be monitored for bleeding and assessed for concomitant use of fish oil, vitamin E, flaxseed, and other over-the-counter and prescription medications known to affect platelet function (Table 4). To minimize bleeding risks in patients receiving ibrutinib therapy, the risks and benefits of concomitant use of antiplatelet and anticoagulant medications should be weighed. In addition, withholding ibrutinib should be considered for at least 3 to 7 days before and after surgery, depending on the risk of associated bleeding (Pharmacyclics, 2014).

**Table 4:**
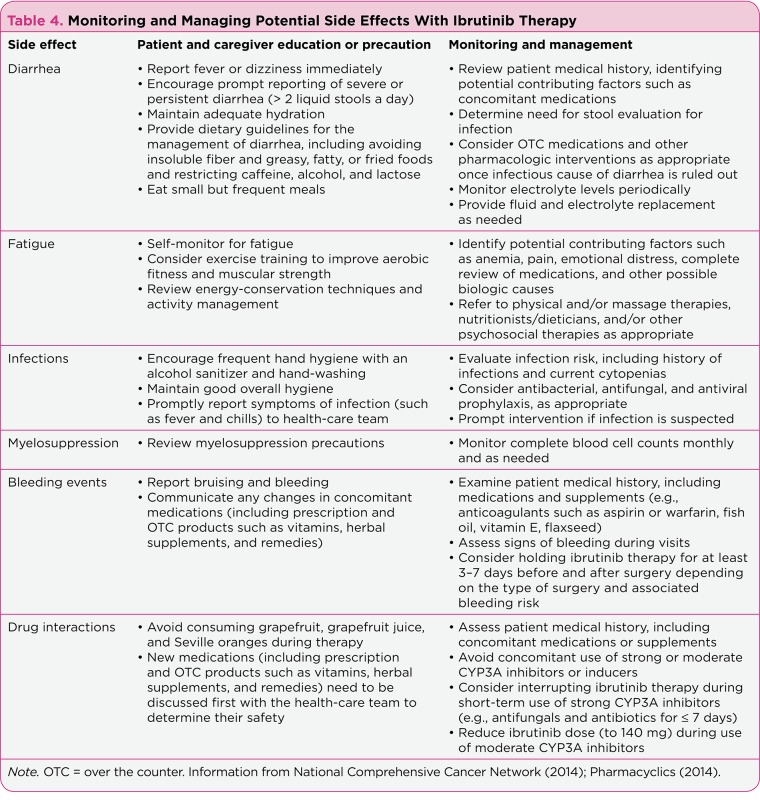
Monitoring and Managing Potential Side Effects With Ibrutinib Therapy

Grade ≥ 3 infections occurred in at least 25% of patients with MCL and 24% of patients with CLL who were treated with ibrutinib (Byrd et al., 2014a; Pharmacyclics, 2014). Infectious events included sepsis and bacterial, fungal, or viral infections, which have been associated with hospitalization and death. Antibiotic and antiviral prophylaxis may be indicated in select patients. Administration of intravenous immunoglobulin G (IVIG) in patients with hypogammaglobulinemia (for recurrent infections and if IgG levels < 500 mg/dL) can minimize the possible development of infectious complications (National Comprehensive Cancer Network, 2014). The importance of frequent hand hygiene should be stressed. Patients and caregivers should be instructed to report symptoms of infection, such as fever and chills, promptly for appropriate assessment and treatment (Table 4).

In the RESONATE trial, atrial fibrillation was noted in 10 patients in the ibrutinib group, leading to the discontinuation of ibrutinib in one patient. One patient developed atrial fibrillation in the ofatumumab group. Potential reasons for atrial fibrillation occurring among patients receiving ibrutinib are being explored (Byrd et al., 2014a).

Treatment-emergent grade 3 or 4 cytopenias were reported in 41% of patients with MCL who were treated with ibrutinib (29% neutropenia, 17% thrombocytopenia, 9% anemia). Grade 3 or 4 cytopenias also occurred in the RESONATE trial with CLL patients and was similar between the ibrutinib and ofatumumab arms (ibrutinib: 16% neutropenia, 6% thrombocytopenia, 6% anemia; ofatumumab: 14% neutropenia, 4% thrombocytopenia, 8% anemia). Monthly laboratory evaluation for complete blood cell counts is recommended (Byrd et al., 2014a; Pharmacyclics, 2014).

**Side-Effect Management**

Diarrhea is the most frequently reported adverse event associated with ibrutinib, affecting 50% of patients with MCL and 48% of patients with CLL treated in clinical trials; the majority of these cases were grade 1 or 2 events, and ibrutinib therapy was not discontinued because of diarrhea (Byrd et al., 2013; Byrd et al., 2014a; Wang et al., 2013). Moreover, colitis was not reported in the aforementioned clinical trials with ibrutinib.

Patients and caregivers should anticipate diarrhea, and they should be informed of appropriate dietary and pharmacologic interventions, including the importance of aggressive oral hydration (Table 4). Electrolytes should be monitored for imbalances and treated appropriately. Patients should be instructed to contact their health-care team if diarrhea persists.

Fatigue, mostly grade 1 or 2, was reported in 41% of patients with MCL and 28% of patients with CLL treated in clinical trials with ibrutinib (Byrd et al., 2014a; Wang et al., 2013). Fatigue is frequently reported by patients with cancer, with a profound negative impact on patient outcomes, including symptom distress and decreased quality of life (Borneman, 2013; Mitchell, Beck, Hood, Moore, & Tanner, 2007).

Nonpharmacologic interventions such as low-impact exercise have been shown to improve cancer-related fatigue, and evidence supports approaches such as education regarding energy conservation and activity management (Borneman, 2013; Mitchell et al., 2007). Referrals for physical, occupational, and psychosocial therapies may be beneficial (Borneman, 2013; Table 4).

## Implications for Clinical Practice

Advanced oncology practitioners play a critical role in providing guidance and education to patients and caregivers on the successful self-administration of ibrutinib and in explaining the mechanism of action of this novel oral agent. Patients have an increased responsibility for self-administered therapies, and serious consequences can occur with poor management or nonadherence, including severe side effects, disease progression, or even death (Yagasaki & Komatsu, 2013).

Patients with MCL and CLL tend to be older and may face unique challenges, such as diminished physical and cognitive capabilities, increased risk of drug interactions, polypharmacy, and adverse events related to comorbid conditions. Anticipating patient needs to allow for early intervention and proactive care is essential. Provider support also includes coordinated effort and close collaborations with other members of the health-care team, as well as provision of psychological and emotional support to further encourage patient empowerment and self-management (Yagasaki & Komatsu, 2013).

## Conclusion

BCR signaling pathways present a new class of promising therapeutic targets. Ibrutinib is a first-in-class oral BTK inhibitor. Approval of ibrutinib offers a novel, effective oral treatment option in patients with relapsed or refractory MCL and CLL, as well as for both treatment naive and relapsed CLL with del 17p. Ibrutinib has a favorable toxicity profile and can be safely administered in a heavily pretreated and older adult population. This single agent induced a high response rate and durable remissions in both MCL and CLL (Byrd et al., 2013; Byrd et al., 2014a; Wang et al., 2013). Ibrutinib offers a new, effective treatment option for patients who typically have a poor prognosis and few other treatment options available.

**Acknowledgments**

The authors sincerely thank Pam Commike, PhD, and Patricia Y. Ryan, PhD, RN, AOCN®, for their helpful discussions. We also thank Pharmacyclics for review of the manuscript for data accuracy and supporting the medical writing assistance of Robert Rydzewski, MS, CMPP.
